# Thinking politically about intersectoral action: Ideas, Interests and Institutions shaping political dimensions of governing during COVID-19

**DOI:** 10.1093/heapol/czae047

**Published:** 2024-11-18

**Authors:** Fran Baum, Connie Musolino, Toby Freeman, Joanne Flavel, Wim De Ceukelaire, Chunhuei Chi, Carlos Alvarez Dardet, Matheus Zuliane Falcão, Sharon Friel, Hailay Abrha Gesesew, Camila Giugliani, Philippa Howden-Chapman, Nguyen Thanh Huong, Sun Kim, Leslie London, Martin McKee, Sulakshana Nandi, Lauren Paremoer, Jennie Popay, Hani Serag, Sundararaman Thiagarajan, Viroj Tangcharoensathien, Eugenio Villar

**Affiliations:** Stretton Health Equity, University of Adelaide, Adelaide, SA 5005, Australia; Stretton Health Equity, University of Adelaide, Adelaide, SA 5005, Australia; Stretton Health Equity, University of Adelaide, Adelaide, SA 5005, Australia; Stretton Health Equity, University of Adelaide, Adelaide, SA 5005, Australia; Research Unit, Médecine pour le Peuple, Rue du Comte de Flandre 25, Brussels 1080, Belgium; Center for Global Health, Oregon State University, Corvallis, OR 7331, USA; University of Alicante, Ctra. San Vicente s/n, 03690 San Vicente del Raseig, Alicante, Spain; LLM, University of São Paulo, Brazil, Av. Dr. Arnaldo, 715–21- Cerqueira César, São Paulo, SP 01246- 904, Brazil; Australian National University, Canberra, ACT 2600, Australia; Research Centre for Public Health, Equity and Human Flourishing, Torrens University Australia, Adelaide, SA 5000, Australia; College of Health Sciences, Mekelle University, Mekelle 231, Ethiopia; Faculty of Medicine, Universidade Federal do Rio Grande do Sul, Rua Ramiro Barcellos, 2400 CEP 90035-003, Porto Alegre, Brazil; Department of Public Health, University of Otago, PO Box 7343 Newtown, Wellington 6042, New Zealand; Faculty of Social Science and Behavior, Hanoi University of Public Health, 1A Duc Thang Road, Duc Thang Ward, North Tu Liem District, Hanoi, Vietnam; People’s Health Movement, 36 Sadang-ro 13-gil, Dongjak-gu Seoul 07004, South Korea; School of Public Health, University of Cape Town, Falmouth Rd, Observatory, Cape Town 7925, South Africa; London School of Hygiene & Tropical Medicine, Keppel St., London WC1H 9SH, United Kingdom; Public Health Researcher, Ebertsgade 6, 2300 Copenhagen S, Denmark; Political Studies, University of Cape Town, Rondebosch, Cape Town 7700, South Africa; Division of Health Research, Faculty of Health & Medicine, Lancaster University, Bailrigg, Lancaster LA1 4YW, United Kingdom; Department of Internal Medicine, University of Texas Medical Branch (UTMB), 301 University Blvd., Galveston, TX 77555, USA; People’s Health Movement Adjunct Faculty, International School of Public Health, Jawaharal Institute of Postgraduate Medical Education and Research, 30, Thiru Nagar, Puducherry 605010, India; International Health Policy Programme, Ministry of Public Health, Nonthaburi 11000, Thailand; Facultad de Salud Pública y Administración, Universidad Peruana Cayetano Heredio, San Martín de Porres 15102, Peru

**Keywords:** Politics, social inequality, social determinants, equity, evidence-based policy, governance, COVID-19, health policy, institutional theory, policy analysis

## Abstract

Our paper examines the political considerations in the intersectoral action that was evident during the SAR-COV-2 virus (COVID-19) pandemic through case studies of political and institutional responses in 16 nations (Australia, Belgium, Brazil, Ethiopia, India, New Zealand, Nigeria, Peru, South Africa, South Korea, Spain, Taiwan, Thailand, Vietnam, UK, and USA). Our qualitative case study approach involved an iterative process of data gathering and interpretation through the three Is (institutions, ideas and interests) lens, which we used to shape our understanding of political and intersectoral factors affecting pandemic responses. The institutional factors examined were: national economic and political context; influence of the global economic order; structural inequities; and public health structures and legislation, including intersectoral action. The ideas explored were: orientation of governments; political actors’ views on science; willingness to challenge neoliberal policies; previous pandemic experiences. We examined the interests of political leaders and civil society and the extent of public trust. We derived five elements that predict effective and equity-sensitive political responses to a pandemic. Firstly, effective responses have to be intersectoral and led from the head of government with technical support from health agencies. Secondly, we found that political leaders’ willingness to accept science, communicate empathetically and avoid ‘othering’ population groups was vital. The lack of political will was found in those countries stressing individualistic values. Thirdly, a supportive civil society which questions governments about excessive infringement of human rights without adopting populist anti-science views, and is free to express opposition to the government encourages effective political action in the interests of the population. Fourthly, citizen trust is vital in times of uncertainty and fear. Fifthly, evidence of consideration is needed regarding when people’s health must be prioritized over the needs of the economy. All these factors are unlikely to be present in any one country. Recognizing the political aspects of pandemic preparedness is vital for effective responses to future pandemics and while intersectoral action is vital, it is not enough in isolation to improve pandemic outcomes.

Key Messages

- Politics affects the ways in which countries responded to the COVID-19 pandemic.
- Institutional factors, ideas including neoliberalism, and attitudes towards science and the interests of political leaders and civil society shape the ways in which countries responded to the pandemic.
- Political will, multisectoral responses, citizens’ trust and civil society support for effective pandemic responses were all important to effective responses.
- Political aspects of pandemic preparedness and action are vital for effective responses to future pandemics.

## Introduction

On 11 March 2020, the World Health Organization (WHO) declared the SARS-CoV-2 virus (COVID-19) a pandemic. By 13 July 2023, roughly 691 million were diagnosed with COVID-19, and 6.9 million had died with COVID-19 as a registered cause (Worldometer, 2023). The virus spread swiftly, with nations grappling with limited knowledge regarding transmission, treatment and prevention. The pandemic had implications across multiple government sectors, including treasuries, education and employment. Pandemics require an intersectoral approach as the emergency has implications for all sectors of society. How governments and international agencies responded became as much a political as a health issue, proving an ideal opportunity to investigate the interface between public health and politics, and the ways in which action across sectors is vital.

Historically, public health decisions have political roots. Virchow in the 19th century linked the spread of infectious diseases, such as typhus, cholera and tuberculosis to living conditions (Waitzkin, 2006). Similarly, in 1848, Engels connected health to living standards (Engels, 2009). The contemporary focus on the commercial determinants of health similarly exposes the health impacts of political practices of tobacco, fast food, alcohol and other corporations (Gilmore *et al*., 2023). These cases also demonstrate the extent to which public health problems are created in multiple sectors and need cross-sectoral responses. While the political and intersectoral nature of the COVID-19 response was clear from the outset, comprehensive analyses comparing political influence across high-, middle- and low-income countries are rare. Most studies target specific nations or particular aspects of politics (Parker and Ferraz, 2021). For instance, Cairney (2021) found UK politicians were selective in heeding scientific advice, while Yuen *et al*. (2021) contrasted responses in Hong Kong and Singapore, highlighting the importance of trust in government, as did Altiparmakis *et al*., (2021). Fofana’s (2021) study from Africa emphasized decolonized approaches to the pandemic, while Howden-Chapman *et al*., (2023) compared the effects of different governance regimes in 15 cities. In this paper, we examine the politics of COVID-19 through case studies of 16 nations (Australia, Belgium, Brazil, Ethiopia, India, New Zealand, Nigeria, Peru, South Africa, South Korea, Spain, Taiwan, Thailand, Vietnam, UK and USA), aiming to uncover key factors for a successful intersectoral public health response to pandemics.

## Theoretical tools

The political influence on public health is often hidden and not immediately evident (Tesh, 1988). Considerable literature exists on frameworks outlining the conditions for effective intersectoral action for health (Harris *et al*., 1995; Public Health Agency of Canada and World Health Organization, 2008; Shankardass *et al*., 2012), including from the World Health Organization in relation to intersectoral action (Brown *et al*., 2014) and Health in All Policies (World Health Organization, 2023). While these documents do recognize the importance of political will to effective action they say very little about the actual workings of the political processes that happen during intersectoral collaboration. Consequently, in this paper, we draw on Hall (1997)’s three Is framework to underpin our discussion of the politics of intersectoral action during the COVID-19 pandemic. Institutional theory suggests that policies emerge from clashing actor interests and ideas within institutional structures, i.e. ‘the ways in which those involved with the issue understand and portray it’ (Shiffman and Smith, 2007). Pandemics create tensions between sectors as is clearly seen between health departments that want to restrict movement, face-to-face work participation and socialization, and economic sectors that want to minimize restrictions and ensure the economy is as little affected as possible. Politics can hinder evidence usage in policy-making (Pawson, 2006). Smith (2013) notes that ideology can be a powerful factor in determining policy but that the acceptance of new ideas can be impeded by institutional and organizational processes. Different philosophies and conceptualizations underpinning pre-existing approaches to governance shaped responses to the pandemic, for example, neoliberalism, biomedicine, equity, human rights and recognition of the social determination of health. These ideas are strategically framed by actors (individuals or groups) in pursuit of their interests or ‘tangible motives’ (Hall (1997), in order to mould others perceptions (Townsend *et al*., 2019) often resulting in dominant frames becoming accepted truths (Benford and Snow, 2000) (Koon *et al*., 2016). The extent of public trust in government responses, critical to the politics of the epidemic (Bollyky *et al*., 2022), also depends on factors in each of the three Is. Table 1 indicates the questions raised about political aspects of intersectoral action for health within the three Is framework. These questions guide our results section by considering the politics of pandemic responses through a political and intersectoral lens.

**Table 1.** The three Is and political aspects of intersectoral action for health

Issue	How the is matter to political aspects of intersectoral action for health
Institutions (Laws, structures, economic structures)	What is the governance structure of the country? What is the institutional history of the country such as colonialism or disruption to structures? Are there laws in place to support public health responses? Are there well-established and functioning public health advisory structures? Are there established governance mechanisms for intersectoral action? What are the existing global and national structural inequities that shape abilities to respond to public health issues?
Ideas (what ideas and cultural assumptions guide action and how are they framed)	What type of economic paradigm is prevalent and how does this influence public health policy and actions? How does the local political climate help or hinder the adoption of public health ideas? What political ideas challenge the scientific consensus? How are dissenting views from this consensus expressed? Are human rights recognized as valid? Do governments predominantly adopt individualist or collectivist ideas?
Interests (individuals and groups of actors within and without government who affect policy)	Do all sectors have the same interests? What is the level and impact of public trust? How is political will for public health action generated and maintained? Who are the different sector stakeholders with an interest in influencing decisions about public health action? How do changing political circumstances enable (or constrain) these different interests from influencing public health policy? Is there an acceptance of the value of citizen participation? Is the community involved in decisions? Is the voice of civil society heard? What is the relationship between civil society and government in regard to public health?

### Methods

We employed a comparative qualitative case study approach, involving 16 countries. This design facilitated understanding of how different political systems addressed the pandemic, accounting for each country’s unique historical and political context (Yin, 2018). The application of political theory enabled us to derive general lessons concerning political responses to a pandemic (Ketokivi and Choi, 2014).

Our country selection considered three criteria: COVID-19 performance based on excess death rates from the Global Burden of Disease study; the presence of local researchers knowledgeable about the political response to COVID-19; and a mix of low-, middle- and high-income countries across diverse continents and political regimes.

The Australian research team spearheaded the study and assembled a team of researchers with expertise in the case study countries. Recruitment of country experts was through the authors’ membership of a global health research network (the Punching Above Weight Research Network), civil society organization the People’s Health Movement, and snowballing through the authors research collaborators. Country cases had typically 1–2 contributing experts, with a total of 22 country experts. In most cases, experts were academic or policy researchers, who were residents and/or citizens of the case study country. The Australian research team created a data collection template for each country informed by the literature on COVID-19 outcomes. The template included questions on governing systems, political leadership, COVID-19 performance and the role of civil society. The Australian research team also gathered data on how civil society rated each country on a scale of ‘repressed’ to ‘open’ during the pandemic (CIVICUS, 2021).

We sought a balance between a systematic and consistent approach to data collection across the 16 countries with the necessity to adapt to what was available in each country, and to capture the most important issues in each case study country. Researchers from each country then provided data against the template between July 2021 and March 2023, derived from their expertise and relevant literature. The research team tailored their web and database searches to draw on: academic articles and books; government reports and websites; reports by non-government and international institutions; and media such as online newspaper articles. Grey literature was included because of the rapidly changing nature of the pandemic, and to understand local political and civil society viewpoints usually not included in academic literature. While any study using expert informants has scope for bias in interpretation, we were careful to validate accounts, as far as possible, with other sources of information, including academic and grey literature. The strengths of using expert informants were their knowledge of their country context, and filling knowledge gaps not easily resolved in a web-based literature search. Having country experts was also crucial to allow inclusion of local literature that was not in English. Draft accounts of each case study country, responding to the data collection framework, were developed by the country researchers with feedback from the Australian research team. The literature searches were further updated in early 2023.

The Australian research team collated the 16 case studies and identified the political factors, ideas and interests evident in each case, led by the first author and developed through team discussion. Our goal was not a comprehensive review of each nation’s response, but rather to highlight how political factors and the ideas and interests in different sectors influence a country’s COVID-19 strategy and identify those that contributed to low death rates.

### Results

We begin with each case study country’s COVID-19 performance based on excess deaths. Then, applying the three Is framework, we explore the political dynamics in our case study countries during the pandemic. First, we assess political institutional structures, their pre-existing pandemic response mechanisms, and the laws and systems employed during the crisis. Next, we examine the ‘ideas’ behind responses, evaluating acceptance of biomedical and neo-liberal economic models and the priority given to equity and human rights. We conclude by analysing political actors’ ‘interests’, gauging both political actors and civil society reactions to pandemic measures.

#### COVID-19 performance

Table 2 shows cumulative excess death rates. In 2020, countries with high death rates remained consistent in 2022: Belgium, Brazil, India, Peru, South Africa, Spain, UK and USA. By 2022, these countries recorded over 250 excess deaths per 100 000, with India at 266 and Peru at a staggering 946 per 100 000. The richer countries had older populations and so were more vulnerable to excess deaths.

**Table 2.** Case study countries cumulative excess deaths from COVID-19 in 2020, 2021 and 2022

	Excess deaths per 100 000, 1 December 2020	Excess deaths per 100 000, 1 December 2021	Excess deaths per 100 000, 1 December 2022
Australia	3.90 (3.90–3.90)	8.72 (8.72–8.72)	65.99 (65.99–65.99)
Belgium	157.94 (148.07–187.23)	253.74 (237.88–300.78)	309.16 (289.83–366.45)
Brazil	97.31 (87.45–115.19)	332.45 (301.73–391.65)	372.80 (338.45–439.14)
Ethiopia	40.60 (26.74–62.34)	160.76 (101.90–246.84)	179.96 (114.07–276.31)
India	76.56 (59.95–95.62)	251.97 (196.24–314.70)	266.24 207.32–332.30)
New Zealand	0.79 (0.79–0.79)	1.36 (1.36–1.36)	50.18 (50.18–50.18)
Nigeria	24.93 (16.93–34.84)	62.45 (42.41–87.25)	66.17 (44.94–92.46)
Peru	398.32 (300.48–526.54)	879.60 (663.55–1162.74)	946.66 (714.15–1251.39)
South Africa	110.84 (85.80–145.57)	461.38 (357.12–605.96)	525.30 (406.60–689.91)
South Korea	1.04 (1.01–1.31)	7.16 (6.95–9.05)	59.11 (57.35–74.79)
Spain	169.08 (149.97–194.04)	315.17 (279.61–360.27)	419.55 (372.38–478.29)
Taiwan	0.07 (0.07–0.07)	3.67 (3.67–3.67)	60.92 (60.92–60.92)
Thailand	0.13 (0.11–0.17)	41.9 (32.88–52.78)	66.97(52.54–84.36)
UK	111.94 (111.20–113.23)	254.04 (252.25–257.09)	313.69 (311.37–317.68)
USA	106.54 (94.27–122.83)	304.72 (269.16–351.46)	417.54 (369.03–481.19)
Vietnam	0.13 (0.09–0.19)	50.87 (33.06–74.02)	86.6 (56.28–126.01)

Source: Institute for Health Metrics and Evaluation COVID-19 projections: https://covid19.healthdata.org/global?view=cumulative-deaths&tab=trend.

By 2022, most other nations had rates below 100 per 100 000, except for Ethiopia, where civil conflict likely inflated numbers. New Zealand had the lowest rate: 50.2 deaths per 100 000.

We also ranked our country cases by their excess death rates for 2020, 2021 and 2022 (see Table 3).

**Table 3.** Country rankings and change in ranking in terms of cumulative excess deaths from COVID-19 in 2020, 2021 and 2022

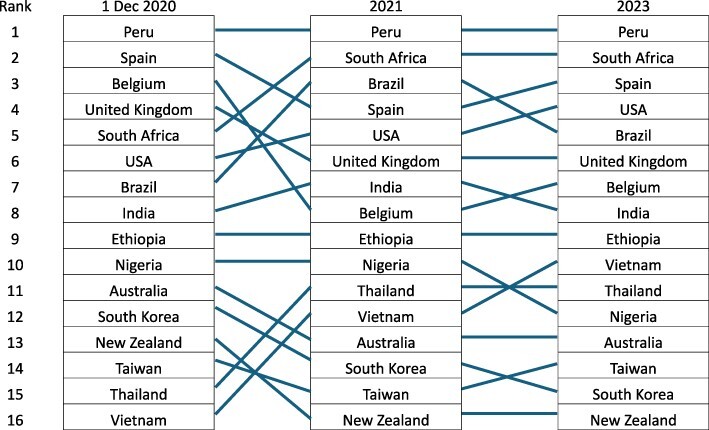

This analysis shows clusters of countries. The better performing cluster—Australia, New Zealand, Taiwan, South Korea, Vietnam—maintained relatively low excess mortality, although in 2022 their rates increased in the Omicron wave. Peru, Spain, South Africa, UK and USA stayed in the worst performing cluster from 2020 to 2022. Ethiopia and India rates remained in the mid-point cluster over time but Brazil joined the worst performing countries in 2022.

### Analysis of the three Is

**Figure 1.**
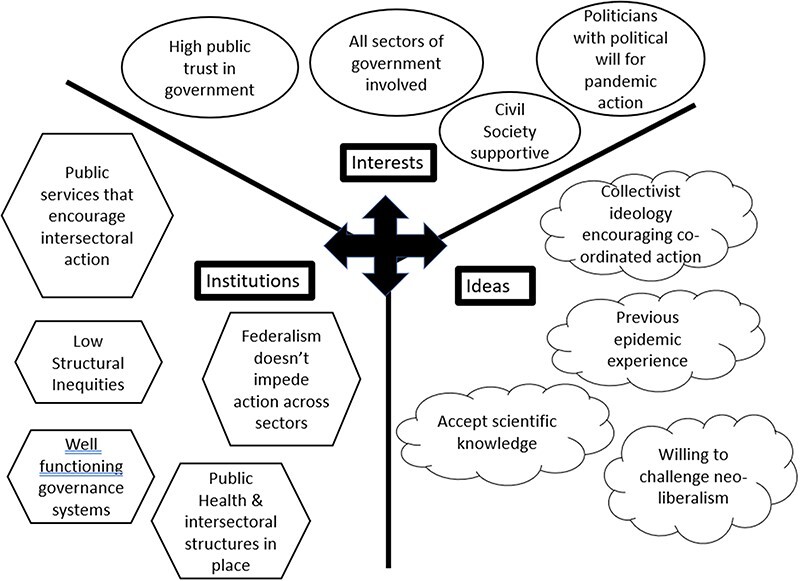
Institutions, ideas and interests which encourage effective COVID-19 responses

Our analysis explored pandemic responses in the case study countries through the lens of the three interconnected Is (see Figure 1). The issues considered within the three Is overlap and while counted within one category often has some relevance for the others.

#### Institutional factors: structures, governance, accountability and constraining factors affecting pandemic responses

Here we examine political systems in each country, especially the influence of federalism on pandemic responses. We then discuss the structures and legislation shaping COVID-19 reactions and consider differential vaccine access as an example of the influence of global economic inequities.

#### Political tensions and macroeconomic policies

Table 4 portrays each country’s political system at the start of the pandemic. GDP varied significantly, and as discussed later affected a country’s capacity to respond. Most nations are classified as parliamentary democracies but some experienced significant political tensions during the pandemic, which shaped their response efforts. For instance, India, which post-independence opted for a secular polity and a liberal democratic state, has recently embraced a Hindutva ideology, which opposes secularism, and has reduced civil liberties.

**Table 4.** Political and economic profile of case study countries

Country	Type of Political regime (March 2020)	2019 GBD per capita	2019 Income category	Gini index household disposable income	Gini index wealth
Australia	Parliamentary Democracy Right wing	53 320.26904	High income	32.5	0.656
Belgium	Parliamentary Democracy	54 545.15088	High income	26	0.603
Brazil	Parliamentary Democracy Right wing populist	15 258.85083	Upper middle income	47.9	0.849
Ethiopia		2311.704386	Low income	33.5	0.62
India	Parliamentary Democracy Right wing fundamentalist Hindutva	7034.217224	Lower middle income	49.7	0.832
New Zealand	Parliamentary Democracy Left wing	43 952.54843	High income	32.8	0.672
Nigeria	Parliamentary Democracy	5348.339797	Lower middle income	42.7	0.809
Peru	Parliamentary Democracy	13 380.36442	Upper middle income	45	0.788
South Africa	Parliamentary Democracy	12 999.12026	Upper middle income	62.5	0.806
South Korea	Parliamentary Democracy	43 028.89635	High income		0.606
Spain	Parliamentary Democracy	42 214.13039	High income	33.2	0.694
Taiwan	Parliamentary Democracy		High income	29	0.751
Thailand	Military Coup Parliament suspended	19 228.29468	Upper middle income	39.7	0.846
U K	Parliamentary Democracy	48 709.70114	High income	33.5	0.746
USA	Parliamentary Democracy	65 118.35833	High income	38.7	0.852
Vietnam	One Party Communist State	8374.444328	Lower middle income		0.761

Sources: Type of political regime author assessment; GDP per capita and income category: World Bank Databank: https://databank.worldbank.org/home.aspx; Gini index household disposable income: Standardized World Income Inequality Database: https://fsolt.org/swiid/Gini index wealth; Credit Suisse Global Wealth Databook 2019: https://www.credit-suisse.com/media/assets/corporate/docs/about-us/research/publications/global-wealth-databook-2019.pdf

Pre-COVID, a historical commitment to the welfare state in many case study countries had weakened under strongly pro-market right wing governments hostile to public services and public management as in Brazil, India, UK and the USA. In these countries, the balance of private and public services appeared more crucial than within government intersectoral responses. Vietnam’s one-party communist state has seen rapid economic development and political stability and proved to have a coordinated and effective pandemic response in the absence of these tensions. In South Korea, a reactionary right-wing alliance of the opposition party, Korean Medical Association (KMA) and right-wing news media politicized the pandemic and stuck to ‘Wuhan Pneumonia’ as the name of the disease showing political distrust of China (People’s Health Movement Korea, 2020b). Some nations faced political crises prepandemic. Thailand witnessed a military coup; Peru grappled with political unrest and economic protests. Ethiopia plunged into civil war in 2020, making pandemic management challenging. South Africa experienced civil disruptions impacting pandemic management. July 2021 saw unrest in Gauteng and KwaZulu Natal provinces including theft from pharmacies, compromising COVID-19 vaccine stocks and patient records (Makina, 2021), disrupted supply chains, hindering oxygen and food delivery to hospitals, national vaccination programmes, and the burning of a CIPLA-owned generic medicine facility (Malan, 2021).

Table 5 displays World Governance Indicators for 2021. Governance quality correlates with countries’ GDP. Nigeria, facing terrorism and corruption (Yagboyaju and Akinola, 2019), along with Ethiopia’s civil war, rendered them ill-prepared for the pandemic. Yet countries with more stable governance like the USA, UK, Spain and Belgium had high death tolls.

**Table 5.** Case study countries scores for World Governance Indicators 2021 (scores are from 0 to 100 and higher scores correspond with better governance)

	Government effectiveness	Regulatory quality	Control of corruption	Rule of law	Voice and accountability	Political stability and absence of violence/terrorism
Australia	92.79	98.56	94.71	92.79	94.20	74.06
Ethiopia	31.25	16.83	39.42	29.33	20.29	4.25
New Zealand	88.94	97.60	99.04	98.08	99.03	96.70
Nigeria	14.42	15.87	14.90	21.15	30.43	6.13
South Korea	90.87	83.65	77.40	84.62	77.78	67.45
Taiwan	91.83	91.35	85.10	87.98	86.47	72.17
Thailand	60.58	56.73	35.10	55.77	27.05	27.36
Vietnam	62.02	37.98	47.12	48.56	13.04	44.81
Belgium	83.17	87.02	89.42	88.46	90.34	66.04
Brazil	35.10	48.08	34.62	42.31	56.04	28.77
India	62.50	49.52	46.63	51.92	51.69	24.53
Peru	41.35	55.29	29.33	33.17	53.62	32.08
South Africa	51.92	50.00	55.77	56.25	72.46	21.70
Spain	78.85	74.04	76.44	78.85	80.19	64.62
UK	86.54	90.87	93.27	89.42	92.75	62.74
UStA	88.46	90.38	83.65	88.94	74.88	47.64

Source: World Bank Worldwide Governance Indicators: https://info.worldbank.org/governance/wgi/Home/Reports

An analysis of Spain’s weak response concluded that ‘crony capitalism’ in Spanish governments prior to 2008 had weakened institutions and created an inadequate policymaking process (Royo, 2020a). Royo also notes that the Spanish government rarely encouraged public input into decision-making (likely to have impacted on trust) and failed to engage the opposition in a timely and constructive way. Peru exemplifies the convergence of various factors leading to an ineffective pandemic response (Gianella *et al*., 2021). Structural socio-economic and health inequalities, aggravated by successful macroeconomic but socially regressive neoliberal policies, and widespread corruption, severely deteriorated public health and education infrastructure and frequent changes of authorities (5 Presidents and 12 Ministers of Health during the COVID-19 pandemic) eroded social cohesion and confidence in state institutions in all sectors.

Privatized public health services performed poorly. The UK contracted out ‘test and trace’ system was ineffective (Garg *et al*., 2022); in contrast, the NHS handled vaccine roll out well (UK Parliament: Health and Social Care, 2021). In Australia, weaknesses were quickly revealed in higher death rates in privatized aged care (Bachelard, 2020) and in failed private quarantine security services systems (de Courten *et al*., 2020). In India, private health sector fees rose and the public sector was unable to respond to increased demand (Thiagarajan, 2020; Garg *et al*., 2022).

Governments, mainly in high-income countries, provided economic support to businesses and individuals, highlighting unequal historical advantages enabling social safety nets.

#### Federalism

Federalism’s impact on responses varied. Federal systems can make intersectoral action more complicated because of different responsibilities at different levels. In Australia, the relative autonomy given to states and territories in leading responses was associated with lower caseloads and death. A National Cabinet was instituted to coordinate activity across the federation and between different sectors. In other contexts, tensions in federal–state relationships affected their ability to manage COVID-19 including in Spain’s autonomous regions. In Brazil, despite clear health laws locating responsibilities with federal government, responsibility for pandemic management was pushed onto states (Royo, 2020a). In South Africa, variations in how different provinces handled the pandemic, reflected historical patterns of inequality with under-resourced provinces less able to respond effectively (Mpulo, 2020). In India, initially the response was largely driven by the centre, which used draconian powers under the National Disaster Management Act to impose a nation-wide lock-down. As the pandemic progressed, the states could decide on movement restrictions and in 2022 they used the Supreme Court to enforce vaccines as a federal responsibility (Sundararama, 2021). Thailand delegated power to provincial governors supported by inter-sectoral provincial disease control committees which proved effective (Tangcharoensathien *et al*., 2023). In Nigeria conflicting Federal/State pandemic regulations led to political friction as citizens adhering to Federal pandemic regulations were arrested, detained or prosecuted for contravening conflicting state regulations (Ibezim-Ohaeri and Ibeh, 2022). Belgium’s multi-tiered governance also caused a complex, often inefficient response with uncertainty over where responsibility lay (van Overbeke and Stadig, 2020). Conversely, Vietnam’s provincial-level coordination and intergovernmental co-ordination played a key role in their success (Huynh *et al*., 2020). In the UK, devolution resulted in varied approaches, with England seen as less effective than Scotland (Tatlow *et al*., 2021). In the USA, long-standing tension between federal guidelines and state practices played out during the pandemic. While most state governors adopted more protective practices than the federal guidelines, a small number of republican state governors ignored these guidelines, refusing to close non-essential business and reopening their economies despite high case numbers (Knauer, 2020).

#### New/existing structures/legislation established to deal with COVID-19

Pre-existing public health frameworks impacted countries pandemic responses (Baum *et al*., 2021). Such institutional factors are helpful in producing supportive ideas and interests adopting those ideas. Having a national Centre of Disease Control (CDC) was advantageous but did not guarantee success. Taiwan’s CDC, with public health laws updated after the SARS epidemic, enabled quick and effective measures (Lee, 2020).

South Korea had an effective Korea Disease Control and Prevention Agency. In contrast, the USA faltered because the Trump administration had previously weakened scientific infrastructure and deliberately ignored the advice of its long-established CDC (Karlawish, 2020). Similarly, Brazil’s robust public health system was undermined by the President’s denialist rhetoric (Werneck *et al*., 2020). Progressive and conservative parliamentary parties collaborated (under pressure from civil society) to pass a law enabling easier compulsory licencing of medical products needed to respond to health emergencies but Bolsonaro vetoed aspects of this. These conditions contributed to high death rates in both countries (Table 2).

Effective responses depended on intersectoral coordination. South Africa was praised for its cross-sectoral action with commentators noting that in addition to decisive, strong leadership from the President, there was effective co-ordination between ministries including Education, Justice, Health, Trade and Industry, Transport, Public Works and Infrastructure, Finance, Cooperative Governance and Traditional Affairs and International Relations and Cooperation (Nkonki and Fonn, 2020).

Existing legislation also bolstered responses. New Zealand adapted its 2017 Influenza Pandemic Plan to the COVID-19 context. In South Korea, the Infectious Disease Control and Prevention Act 2015 made possible the tracing and information disclosure of COVID-19 patients, although civil society expressed concerns about human rights protection under the amended legislation (Kim *et al*., 2020; People’s Health Movement Korea, 2020a). Taiwan’s comprehensive control measures, built over 16 years since SARS, effectively balanced health concerns and individual rights. Thailand quickly mobilized its Centre for COVID-19 Situation Administration (CCSA) and used its 2015 Communicable Diseases Act to empower provincial leaders (Lee, 2020).

Vietnam responded promptly issuing guidelines by January 2020 and implemented aggressive contact tracing and testing (Khánh *et al*., 2020). In 2020, together with Thailand, they had the lowest death rate and remained in the best performing third of countries over time (Table 3). Nigeria swiftly set-up structures to combat the pandemic, including a multi-ministerial Presidential task force, capitalizing on past experiences with polio and Ebola (Abubakar *et al*., 2021). Despite being resource-poor and having internal conflict from insurgents, Nigeria ranked in the best performing third of countries at each time point 2020–22 (Table 3) highlighting the importance of existing infrastructure and past experiences.

#### Existing structural inequities within and between countries

##### Structural inequities between countries

Among the four low- or lower-middle income nations, Vietnam and Nigeria fared well, while Ethiopia and India had higher death rates but none of these countries were in the worst performing cluster (Table 3). Wealthy nations like the USA, UK, Spain and Belgium were primarily ranked in the worst performing third of countries (Table 3), whereas Australia and New Zealand maintained low death rates. These wealthy countries all had older populations who were more susceptible to death from COVID-19, so while this contributed, countries responses were likely to have been a more significant factor.

When vaccines became available, people in poorer countries had less access to vaccines than those in wealthier countries as has occurred with other medicines (Tenni *et al*., 2022). Nigeria, Ethiopia and South Africa achieved low vaccination rates, while richer countries secured vaccines in amounts often in excess of need (Yamey *et al*., 2022). This is shown starkly in the case of Nigeria where only 2.5% of its population had two doses by early 2022 and 29% by early 2023. In contrast, 86% of the South Korean population had two doses by January 2022. Vietnam is an outlier in terms of a low-middle income country, where 87.5% of its population had two doses by early 2023 (Worldometer, 2023). India had capacity to manufacture vaccine and after the development of an indigenous vaccine, achieved a reasonable coverage, in part as a result of civil society lobbying for a universalist policy.

Internal governance regimes had some impacts on access to vaccines. Peru and South Africa faced delayed procurement, while Australia and South Korea began slowly but finally performed well. However, the primary drivers of vaccine inequities were global economic inequities and the power of trans-national corporations, which dictated distribution. The retention of intellectual property (IP) rights by vaccine manufacturers, primarily from the USA and Europe, inflated prices (Gold, 2022), demonstrating how trade regimes privileged TNCs profit protection over global health. Despite South African and Indian leadership at the World Trade Organization and global campaigns to waive IP rights, obstacles remained intact. COVAX, designed to help lower-income nations access vaccines, prioritized pharmaceutical companies’ interests and did not involve governments in decision-making (Katz *et al*., 2021).

##### Structural inequities within countries

South Africa’s response to the pandemic was challenged by existing inequities, a result of apartheid’s legacy and reflected in its high Gini co-efficient (Table 4). The nation grappled with high unemployment, an unstable economy, informal employment and inadequate worker protections (Charles, 2020). Overcrowded townships intensified the virus’s spread and a lack of community voices in statutory structures hampered the COVID-19 response. Moreover, community health workers, pivotal in such scenarios, were erratically incorporated (Hlatswayo, 2021). This was also the case in India, Brazil and Peru (de la Puente, 2021). While all these countries had pre-existing extreme socioeconomic and health inequities, Peru’s performance during the pandemic may have been worse because its social security nets were not as strong as those in Brazil and South Africa.

In Belgium, people >65 years of age from lower income or educational backgrounds had higher mortality rates (Decoster *et al*., 2020), and COVID-19 cases were more frequent in low-income neighbourhoods (Meurisse *et al*., 2022). England’s most disadvantaged groups saw mortality rates quadruple compared to their affluent counterparts (Suleman *et al*., 2021). Australia’s socioeconomically disadvantaged and migrants faced higher death risks (Australian Institute of Health and Welfare, 2021), while in South Korea, lower socio-economic groups were more likely to contract the virus (Oh *et al*., 2021). In the USA, racial disparities became evident, with Black, Hispanic and Indigenous populations facing greater risks than white people and racial inequities were commonly mediated by economic factors (Bruce *et al*., 2022; Ndugga *et al*., 2022). This was also the case in the UK where chronic racism has been shown to have played a role (Nazroo and Bécares, 2021).

**Ideas: assumptions driving pandemic political and intersectoral responses**

Hall (1997) argued that ideologies shape policy responses. This is evident in our country case studies. Three ‘idea’ elements were associated with more successful COVID-19 responses: the questioning of neo-liberalism, the fostering of collective values and political actors’ embrace of science.

#### Questioning neo-liberalism

During the pandemic, a notable shift was observed as even governments that staunchly championed neo-liberal economic ideologies recognized these frames were inadequate in the face of the crisis. High-income countries (Australia, New Zealand, UK and USA) implemented extensive welfare and business support measures and while these supported the economy they also changed norms relating to state spending. Australia, for instance, doubled welfare payments for unemployed individuals from April 2020 to March 2021. Even lower-income nations with limited resources provided aid. Brazil, Ethiopia, Thailand, Peru, South Africa and Vietnam initiated various cash transfers and support schemes. This widespread, swift governmental support suggests a rapid emergence and adoption of new norms. These are all examples of intersectoral action for health and demonstrate the importance of social security policy in a pandemic. A review of the change in social security policy in Australia found that the increased payments meant people could better meet their basic needs, improve their physical and mental health, increased labour market engagement and led to recipients engaging in unpaid productive work (Klein *et al*., 2022). In Brazil, cash transfer payments improved social distancing and reduced rates of contracting COVID-19 (de Leon *et al*., 2023).

Prior adherence to neo-liberal policies, especially privatization of health and care services in countries like Peru, UK and Belgium, appeared to have hindered their pandemic response (Enríquez and Fraga, 2021). Spain had also adopted the neo-liberal policies which had weakened its ability to make an adequate response, while in some low and middle-income countries, the pandemic exposed the shortcomings of public–private care models. In contrast, Thailand’s universal system enabled it to cover full costs of care for everyone, invaluable to its pandemic response.

#### Individual or collective values and ideas

Individualistic norms have been argued to be a barrier for effective COVID responses because they cause governments to be reluctant to introduce restrictive measures (Jiang *et al*., 2022). We similarly found that the strong link between neoliberalism and individualism (Baum and Fisher, 2014) meant countries favouring neoliberalism often leaned towards individualistic responses, as seen particularly in the USA, UK, Spain, Brazil and Peru, all of which were at or near the bottom of the performance rankings (Table 3).

In contrast, communist Vietnam, emphasizing collective values, quickly implemented public health measures from past epidemic experiences. They fostered public unity and their slogan ‘each citizen is a warrior to fight COVID-19’ underscored social solidarity. Similarly, Taiwan’s affordable ‘mask miracle’ (Chi, 2020) highlighted its societal cohesion. Universal public health systems in countries like Australia, UK, New Zealand and Taiwan supported coordination, although these systems are being undermined by pressures to privatize that pre-date COVID-19, as is evident in the UK (Pollock, 2006). The USA and other countries with primarily privatized services, reflecting individualistic values, struggled with pandemic coordination.

#### Role and acceptance of scientific knowledge

The pandemic highlighted the ways in which science is a social practice as well as a knowledge-revealing one and is far from value free (Baskin, 2020). Some initial disagreement among scientists was predictable given the virus was novel but as knowledge about COVID-19 evolved, scientific advice become more consistent. Countries in which there was little debate over the science were more easily able to engage all sectors in pandemic measures. The extent to which this advice was accepted by political leaders and citizens varied, significantly influencing pandemic responses. Countries like South Korea, Taiwan and Vietnam, with past epidemic experiences, quickly adopted pandemic measures based on scientific evidence (Chua *et al*., 2021). Taiwan had a Scientific Advisory Council to guide policy decisions. Although South Africa and Nigeria used scientific advice initially, its use waned over time. US President Trump and Brazil’s Bolsonaro often disregarded or contested scientific consensus, undermining preventive measures and promoting unverified treatments. For example, Trump claimed the antimalarial drugs chloroquine and hydroxychloroquine, the antibiotic azithromycin, ‘disinfectant injections’ and ‘UV light’ as effective treatments despite the lack of evidence (Lasco, 2020). His administration consistently provided inaccurate information about the value of masks, social distancing and washing hands. Both Trump and Bolsonaro attacked the WHO, undermining their global authority (Sott *et al*., 2022). Such denial of science by Presidents made action in all sectors, not just health, less likely. In South Africa, the use of expert advice was bedevilled by secrecy and delays, which hampered its pandemic response (Richter *et al*., 2022).

In Peru, policy failures, such as reliance on inaccurate tests and a lack of any contact tracing and communication campaigns, reflected a disregard for scientific advice (Jaramillo and López, 2021). The UK government claimed to be ‘following the science’, but analysis indicates that the UK used scientific guidance selectively (Cairney, 2021). In response, concerned academics established an Independent Scientific Advisory Group for Emergencies (SAGE) to monitor the government’s adherence to scientific advice.

### Interests of political, bureaucratic and civil society actors: economy, trust, civil society and community engagement

The pandemic affected all sectors of society so that each government department brought their sectoral interests which could clash. This was most clearly seen in the different interests of finance departments and health agencies where the people’s health was not always put before the needs of the economy.

In terms of the interests of politicians, vis-a-vis the population trust and effective communication were vital. The Carnegie Endowment for International Peace highlighted how some populist leaders such as Bolsonaro and Trump governed through polarization during the pandemic (Carothers and O’Donohue, 2020), so diminishing trust in public health measures and science (Eyewitness News, 2020). The UK’s pandemic response was also influenced by leadership dynamics. Prime Minister Boris Johnson missed crucial emergency meetings and prioritized loyalty in his ministerial appointments over relevant experience. Johnson’s government’s response to care homes was judged illegal (McKee, 2022).

Trust was crucial for people to adhere to pandemic measures amid fear and uncertainty. Table 6 reveals varied trust in governments and in ‘most people’ across countries.

**Table 6.** Trust in government and most people (2020)

	Trust in government (%)^a^	Trust in most people (%)^b^
Australia	69.5	54.0
Ethiopia	78.1	11.9
New Zealand	83.7	58.5
Nigeria	23.1	12.6
Singapore		34.0
South Korea	52.8	32.9
Taiwan	65.4	31.0
Thailand	55.6	31.4
Vietnam		27.7
Belgium	54.9	
Brazil	40.6	6.5
India	66.0	16.7
Peru	45.6	5.3
South Africa	50.9	23.5
Spain	48.2	19.0
UK	47.7	46.0
USA	52.5	39.7

a Trust in Government: Share of people who trust their national government, 2020 (Source: Wellcome Global Monitor, (2020).

b Trust in most people: Share of people agreeing with the statement ‘most people can be trusted’, 2022 Data extracted from: https://ourworldindata.org/trust.

In 2020, trust in governments generally surpassed trust in ‘most people’. High-income nations scored highest in both categories, while Nigeria scored the lowest. Notably, Brazil, Peru and Ethiopia exhibited minimal trust in people, with Brazil and Peru experiencing high excess death rates. These data suggest that many low-and middle-income countries faced trust deficits, making it difficult to garner public support. Trust is also vital in intersectoral action (Delany‐Crowe *et al*., 2019), so generalized low trust may inhibit this activity in government and so detract from the pandemic response.

Effective communication and trust in ‘leaders’ and their ‘ideas’ are crucial in alleviating fear and suspicion during crises. Leaders like New Zealand’s Jacinda Arden, Scotland’s Nicola Sturgeon and Wales’s Mark Drakeford were praised for their clear and empathetic messages during the pandemic (Edelman, 2021). Arden’s daily briefings emphasized social unity, promoting the idea that the nation was a ‘team of 5 million’ battling the virus together (Henrickson, 2020). Most countries provided daily updates through various media channels. In South Korea, President Moon Jae-in, a former human rights lawyer especially during the early phase of the outbreak, stressed openness, transparency, and civic engagement. In Nigeria, the NCDC utilized a multi-platform approach to inform the public, while Vietnam and Taiwan made information easily accessible through official channels and mobile apps.

In contrast, many poor performing countries lacked clear communication, leading to public confusion. In the USA, President Trump’s public disagreements with Dr Fauci (his chief medical officer) resulted in mixed messages. The Indian government often used stereotyped messages that lacked appreciation of different people’s circumstances, producing advice on social distancing that was completely impractical for much of the population. A state of emergency announced with less than one day’s notice and no consultation or public preparation, created one of the most stringent and longest lockdowns of any country.

### Role of civil society

Civil society significantly impacted every country’s response to the pandemic, but the extent varied based on historical context and government openness to civil society input. The Human Freedom Index (CIVICUS Monitor, 2021) shows most countries becoming less open between 2019 and 2022 (see Table 7).

**Table 7.** Civil society (CIVICUS) ratings

	CIVICUS Rating 2019	CIVICUS Rating 2022
Australia	Narrowed	Narrowed
Belgium	Open	Narrowed
Brazil	Obstructed	Obstructed
Ethiopia	Repressed	Repressed
India	Repressed	Repressed
New Zealand	Open	Open
Nigeria	Repressed	Repressed
Peru	Obstructed	Obstructed
South Africa	Narrowed	Obstructed
South Korea	Narrowed	Narrowed
Spain	Narrowed	Narrowed
Taiwan	Open	Open
Thailand	Repressed	Repressed
UK	Narrowed	Narrowed
US A	Narrowed	Obstructed
Vietnam	Closed	Closed

Source: CIVICUS Monitor: https://monitor.civicus.org/.

CIVICUS: People Power Under Attack. A Report Based on Data from the CIVICUS Monitor. December 2019. https://civicus.contentfiles.net/media/assets/file/GlobalReport2019.pd

For instance, Nigeria remained rated as repressed with minimal civil society influence, while Spain’s civil freedom deteriorated due to long-standing institutional issues (Royo, 2020a; Samutachak *et al*., 2023). India, consistently rated as repressed, implemented strict pandemic measures, including quarantine zones and severe movement restrictions (Krithi *et al*., 2022) which were largely unsuccessful as containment measures. Contrastingly, Taiwan and New Zealand, both consistently rated as open, had effective pandemic responses. Even in countries rated as repressed local informal support is likely to have been important. For instance, while Thailand was rated as repressed a study suggests that family, community and local networks assisted people in responding to and recovering from the impacts of the pandemic (Samutachak *et al*., 2023). Civil society actions, both positive and unhelpful for pandemic management are examined below together with an assessment of government responses to these actions.

#### Positive actions

Civil society groups commonly provided direct support to communities but their advocacy for specific pandemic measures, notably global vaccine equity greatly influenced political responses across many sectors. South African organizations mobilized support for the TRIPS waiver on vaccine IP, along with civil society in Brazil, India, South Korea, and globally, the People’s Health Movement (People’s Health Movement, 2020). South Africa’s long history of progressive civil society action was particularly important. The South Africa People’s Vaccine Coalition (Msomi, 2021), for example, were strong advocates on the unequal health system, austerity in health care funding, the need to engage and improve employment conditions for community health workers, gendered disparities and intellectual property. Brazil’s Working Group on IP campaigned for vaccine licencing, leading to a bill in July 2021 addressing patent barriers and technology transfer (Working Group on Intellectual Property, 2021).

Civil society action and advocacy also resulted in better support for groups in vulnerable circumstances. In Nigeria, organizations addressed the surge in gender-based violence during COVID-19 and offered free legal helplines for victims of inconsistent lockdown measures (Eribo, 2021). In Peru, civil initiatives tackled health rights denied during the pandemic. In South Africa, spontaneous self-organizing of communities established Community Action Networks to help community-based responses to COVID-19 (van Ryneveld *et al*., 2022), while other groups pressed the state for transparency in vaccine contracts (Health Justice Initiative, 2023) and equity in vaccine allocation (Paremoer and London, 2021).

In South Korea, civil society’s advocacy included watching, criticizing and suggesting alternatives to the government responses and raising issues of privacy or human rights abuses of minorities or vulnerable groups by the government (Civil Society Organizations Network in Korea, 2020; People’s Health Institute, 2020). Trade unions fought for the safety, health and rights of workers at particular risk, including migrant workers, and demanded further strengthening of the public health and care system and reform of the socio-economic system (Ford and Ward, 2021). A ‘Civil Society Task Force’ was launched involving over 500 organizations. In Taiwan, a Fact Check Center countered pandemic misinformation, complementing government efforts (Taiwan FactCheck Centre, 2021) and thanks to civil society advocacy, migrant workers accessed universal healthcare and benefited from wage increases in 2022 (Global Workforce Group, 2022).

The UK’s Good Law Project reviewed procurement decisions during the pandemic, while Independent SAGE showcased professional group self-organization. The JSA (People’s Health Movement in India), alongside the All India Peoples Science Network, published over 30 papers and resolutions targeting decision-makers and the public. Another major intervention was training of village volunteers, drawn from organizations in the field to create public awareness of the disease and address widespread stigma and denial. Legal petitions on food, health and access to medicines were filed by campaigns and networks (Sinha, 2021).

#### Conspiracy theories and anti-vaxxers

Several countries saw protests against pandemic measures, often linked globally through social media and right-wing activist groups. In the US, thousands protested in various cities against perceived infringements on liberties, opposing vaccinations and masks. The US anti-vaxx movement has grown stronger during the pandemic (Carpiano *et al*., 2023). In New Zealand, February 2022 witnessed a 23-day unauthorized occupation of Parliament grounds in Wellington by anti-vaxxers and right-wing extremists (Mitchell and O’Dwyer, 2022). Australia’s state of Victoria, which had extensive lockdowns, faced protests resembling those in New Zealand. As one study in Spain highlighted, political activity by civil society actors representing narrow or ‘uncivil’ groups can reinforce existing economic, social, or cultural cleavages (Rey-García and Royo, 2022). In India voices in the media and society argued that India’s Muslim minority were spreading the disease, fuelling discrimination and even violence (Carothers and O’Donohue, 2020). A high degree of victim-blaming characterized both state and media response (Krithi *et al*., 2022) exacerbating widespread stigma. There was outright denial of an ongoing pandemic in many states of India, possibly a form of coping by people in the most vulnerable circumstances who could not follow any of the recommended personal protections and social restrictions.

#### Government’s reactions to civil society protests

In some countries, notably Taiwan, government and civil society groups collaborated effectively for improved pandemic control, garnering public trust (Lee *et al*., 2020). In Spain, despite civil society being broadly distrustful of government, since the Franco regime there were instances of positive co-operation. For example, the activities of migrant women in informal employment organizing to secure their rights were recognized, although not funded by the Ministry of Health (Martín-Díaz and Castellani, 2022).

In other countries, governments increased crackdowns on civil society during the pandemic. In Nigeria, emergency powers enabled hurried policy decisions with minimal civil society consultation and curtailed civil liberties severely, with drastic actions like shoot-on-sight orders for quarantine violators (Ibezim-Ohaeri and Ibeh, 2022). In Ethiopia, amid a civil war, the government’s actions included media outlet shutdowns, journalist detentions, and NGO operation restrictions, all amidst a backdrop of increasing political and ethnic tensions (Maggie, 2021; Tesfay and Gesesew, 2021). Australia also curtailed protest rights, especially during anti-lockdown demonstrations (BBC, 2021). A sudden lockdown of a Melbourne housing tower led to ‘fundamental breaches of human rights’ (Simons, 2021). In India, press freedom was restricted, and the Modi government imposed limitations on NGO funding while continuing its opposition to civil society organizations advocating civil liberties (Bhattacharya, 2020).

## Discussion

Intersectoral action is embedded in contemporary strategies to improve health and reduce health inequalities and is a central tenet of the UN’s sustainable development goals. The pandemic reinforced the imperative for intersectoral action within and across countries. Previous research and contributors to this volume have identified a multitude of factors hindering or enabling effective intersectoral action but as Buse *et al*. (2022) recently argued in relation to climate change ‘the key to making… intersectoral action work, hinges on thinking politically about it’. Our research contributes to this endeavour through the lens of the COVID-19 pandemic. The comparative analyses of government responses to COVID-19 across 16 country cases highlighted the interacting and dynamic factors that complicate prediction of how well countries handle a pandemic. Our research underscores the significance of historical and present-day contexts shaping political actions and offers insights on optimal conditions for responding to public health threats. It is unlikely that all the positive political factors for good pandemic management will be found in one country as governments work in complex contexts with differing ideologies which constrain responses. We found, as public health theory indicates (World Health Organization, 1986; WHO, 2015), that action in multiple sectors is vital in responding to a pandemic but that while intersectoral action is vital it is not sufficient on its own. Table 8 lists five factors identified as important for effective political responses to a pandemic and maps these to institutions, ideas and interests. We discuss each component below.

**Table 8.** What elements supported by which ideas, interests and institutions are required to support an effective response to pandemics

Elements of effective pandemic response	Ideas	Interests	Institutions
Action in multiple sectors	Health in All Policies principles for effective horizontal governance	Authority from the head of government Incentive for public servants to co-operate	Works best with formal structures for shared governance
Political will and leadership for effective response	Accepting advice on vector and response to disease based on science. Common understanding of the problem and potentially effective solutions	High quality Political Leadership that accepts science. Caring and compassionate. Not populist, appealing to prejudices or questioning science as a means of maintaining power	Stable political system If country is federated good co-ordination between levels of government
Active civil society to which the government is responsive	Acceptance of the important role civil society can play in providing services and advocating for pro-pandemic measures	Civil society leaders attuned to the requirements of a pandemic response and prepared to be strong advocates. Government actors who are responsive to civil society	Structures which allow for government and civil society partnerships Freedom of expression for civil society
Crucial role of trust in a pandemic	Accepting best available evidence	Empathetic leaders generating trust accepting the fluidity of trust in modern society and need to reinforce it regularly	Robust institutions which have the trust of people prior to pandemic
Balance needs of people’s health with those of the economy	Acceptance of role of interventionist state to support businesses and individuals under economic pressures resulting from disease threat	Pressure from business lobby against lockdowns and other public health measures is resisted Believe in supporting people through universal health care and social security systems	Strong social security systems Economic systems that balance the needs of people’s health and that of the economy

### Action in multiple sectors

The pandemic underlined that effective and equitable health promotion and disease prevention relies on actions across multiple sectors (Commission on Social Determinants of Health, 2008). It also made clear that intersectoral action is vital as one of the main responses to the pandemic. Pandemics affect just about every area of people’s lives and as such all sectors are forced to respond. Leadership from the head of government is identified in the Health in All Policies literature (Ollila *et al*., 2013) as critical for effective cross-sectoral action. This was demonstrated in all our countries. Equally important is the need for horizontal governance to coordinate across sectors (Kickbusch, 2010). Leaders in non-health sectors need to be incentivized to consider their sector’s health impact and staff need to be trained in ways of making intersectoral action occur (WHO, 2015). During the pandemic, the health sector provided technical advice across government, mandated mask wearing and social distancing and provided illness care services. School, universities and workplaces had to respond to lock down and education institutions had to teach online. Social security systems had to pivot to provide wider support. Treasuries had to make dramatic changes to budgets to accommodate support for business and individuals. Businesses had to adapt by closing or changing their mode of operation to suit the pandemic circumstances.

But on its own intersectoral action is not enough it also relies on and works synergistically with the other factors we have identified. Thus, effective cross-sectoral action depends on power sharing and trust (Ran and Qi, 2018) and effective political leadership. The pandemic forced cooperation across sectors of government. Structures were set up in most countries to coordinate responses which happened quickly and effectively in most cases. COVID-19 showed us how governments working on an acknowledged crisis can make rapid decisions, adopt different ideas and use both established and new structures to coordinate across sectors rapidly to respond to the threat.

### Political leadership and will

Our research reveals that COVID-19 action is critically dependent on strong political will and leadership. Where leaders understand the role of scientific evidence, communicate in a factual but empathetic manner, without ‘othering’ certain groups, are willing to establish effective cross-government structures to co-ordinate action, are receptive to civil society engagement and refrain from ideological criticism of institutions such as WHO or national public health agencies, then political will could successfully limit disease. Drawing on Post *et al*., (2010) definition of political will, we observed that effective responses require consensus and commitment among the public and decision-makers about best practices for curbing the spread of COVID-19, plus a determination to preference population wellbeing over other considerations.

Effective political leadership can happen in different political contexts. Vietnam, a one-party state, used central planning linked to provincial government to effectively implement successful public health measures. Among liberal democratic states, political responses varied. New Zealand’s effective response in the first 2 years relied on a compassionate leader, following public health scientific advice, and maintaining the trust of the population. Australia’s response had largely bi-partisan support and in the main followed public health advice. In the USA, UK, Spain and Brazil, however, political leadership was less effective. Particularly in Brazil and the USA, scientific advice was frequently questioned at the highest level and basic public health measures, such as mask wearing were heavily politicized. Their COVID-19 stance derived from populist politics and exacerbated an already divided electorate (Carothers and O’Donohue, 2020). Lack of will was largely reflected in those countries stressing political values of individualism. The pandemic highlighted that science does not operate ‘in an elevated sphere of pure reason separate from the social currents around it’ (Ball, 2021). The translation of scientific knowledge into evidence for policymaking depends heavily on the ways in which political actors use it and the extent to which civil society advocates against it, as happened over masks and vaccinations.

Effective responses to the pandemic emerged not just from a reactive government, but from a broad network, including journalists, charities, activists, researchers and politicians. This highlights the need for a robust ecosystem backing pandemic measures. COVID-19 produced many examples of the influence of advocacy coalitions composed of civil society and health professionals, both supporting and opposing public health measures (Sabatier and Jenkins-Smith, 1999). Scientific knowledge can be rejected because of competing interests (especially the economy) and/or the absence of unambiguous evidence on specific measures (Salajan *et al*., 2020). Varying ideas about the importance of public health science can lead to different sectors responding to a pandemic in very different ways. In most countries, tension was evident between public health authorities and treasuries and economic departments.

Given the novelty of COVID-19, dissemination of new knowledge became crucial to bolster political will. WHO’s leadership was pivotal in the global health emergency declaration and country support. Most countries’ political responses were framed in a biomedical (and hospital-based) paradigm. Given this commonality, countries were able to learn from the experiences of each other. The scenes of overwhelmed Italian hospitals seen around the world in the first months of the pandemic were likely to have spurred political will and action on COVID-19. The way in which most governments moved quickly to support individuals and businesses fits the criteria of a norm emergence and cascade (Finnemore and Sikkink, 1998), whereby an idea which had been unacceptable is rapidly adopted, signalling a rapid adoption of new norms Existing international institutional structures, especially the WHO, enabled collaboration and dissemination of evidence which supported the adoption of new norms by national governments.

The COVID-19 pandemic presented a unique political moment. The virus’s sudden and rapid global spread punctuated the equilibrium of governments (Baumgartner *et al*., 2018), forcing them off path dependency and business as usual. All countries saw politicians taking decisions that would have been unthinkable a few months before the pandemic. These measures included restricting individual liberty to reduce likelihood of infections spreading, mandating mask wearing and deferring neo-liberal economic approaches for a willingness to increase government spending on business support and increased welfare payments.

### The ambivalent role of civil society

Civil society from many sectors significantly influenced pandemic politics. Although public health advice necessitated government actions restricting individual rights, Transparency International emphasizes that social movements played a key role in ensuring power and corruption checks and accountability (Vrushi and Kukutschka, 2020). Civil society can fill gaps in government services in low- and middle-income countries such as India, Peru and Brazil, and advocate for the needs of particular populations. However, the rise of populist movements before the pandemic made anti-science sentiments and conspiracy theories popular. Numerous civil society groups, sceptical of public health measures, staged loud protests that have been described as ‘medical populism’, which constructs antagonistic relations between ‘the people’ whose lives have been put at risk by ‘the establishment’ (Lasco and Curato, 2019). The USA and Brazil, with high pandemic-related fatalities, and heavy commitment to individualist values articulated by political leaders, exemplify the dangers of neglecting science.

Pandemic responses have, at times, impinged on freedoms, like the treatment of migrant workers in India or the abrupt lockdowns in Australia and New Zealand, prompting mass protests in some countries. Speed and Mannion (2020) highlight how politicians have manipulated populism in relation to health care systems, which result in multiple social, political and economic inequalities in population health. Health systems prone to populism tend to be poorly resourced, have command and control governance systems and lack public trust. Societies with greater civil society engagement generally benefited in terms of pandemic and political outcomes.

### The role of trust in the politics of the pandemic

Trust is crucial in managing a pandemic (Bollyky *et al*., 2022) just as it is vital in intersectoral action (Delany‐Crowe *et al*., 2019). A study of Health in All Policies found trust bridges the gap between the known and unknown and its existence is important amidst uncertainty (Delany‐Crowe *et al*., 2019). In uncertain situations, trust enables action, particularly where there is perceived to be vulnerability, ambiguity and risk to individuals (Edelenbos and Klijn, 2007). Reliance on expert systems in modern society suggests that if the public trusts biomedical knowledge, they are more likely to heed science-based government advice. Trust can promote cooperation and progress (Carnwell and Carson, 2008) whereas its absence can result in suspicion and non-cooperation, evident in the USA, Brazil and Peru.

Erhardt *et al*., (2021) note the importance of emotion in uncertain times and suggest fear and anger are likely to have different impacts. Whereas fear leads to a ‘rally-round-the-flag’ effect, increasing trust in the government, anger attributes blame for adverse circumstances to government. Our cases suggest trust is fostered when leaders are empathetic and address people’s fear, have access to and follow public health and medical expertise and are seen to put public health ahead of the economy.

However, during crises, people constantly reassess their trust in the government (Giddens, 1991). Initially, lockdowns increased trust in government (Bol *et al*., 2021) but there was less trust over time as shown by increasing public protests. According to Meyerson *et al*., (1996), trust varies in its fragility: some trust relationships are thick and resilient and others are thin and easily lost or withdrawn. Thick and resilient trust was seen most strongly in Taiwan, reinforced by previous experience with epidemics, competent public health institutions and responsive government. In South Korea, trust in the government was low at the start of the pandemic but grew over time associated with proactive responses to the pandemic (Kye and Hwang, 2020). In contrast, in countries where trust in government was low prior to the pandemic such as in Peru and Nigeria, governments were unable to rely on accumulated trust to ensure citizens accepted pandemic control measures. Compromised trust can motivate vigilance, suspicion and an unwillingness to cooperate (Levi and Stoker, 2000) as seen in the rallies against public health measures. Retaining the trust of the population is a vital task for politicians during pandemics and requires an understanding of its fluid, conditional and fragile nature in contemporary society.

### Balancing the needs of the economy with people’s health

Politicians primarily seek re-election (Hall, 1997). This consideration would have been a preoccupation during the pandemic as they grappled with balancing health protection and economic stability. Doing this in all countries was a political juggling act and required politicians to make ethical judgements about the value of people’s health over the state of the economy. Thus, many governments embraced interventionist roles, offering economic aid during lockdowns, thus privileging people’s needs over those of the economy even if it meant departing from neo-liberal policies. However, in all countries, groups disadvantaged by economic circumstances, gender and ethnicity have been the most likely to die (Paremoer *et al*., 2021). Our findings confirm earlier work (Bump *et al*., 2021), showing the extent to which country experiences are shaped by colonialism. Those countries which have experienced extractive colonialism and more recently neoliberal regimes (Nigeria, Peru, South Africa, India), had very high levels of pre-pandemic inequality, resulting in a lack of resources to support their populations during the pandemic including difficulties in acquiring vaccines, PPE and medicines. The ways in which the global economy is stacked in favour of high-income countries, privileging profits of pharmaceutical companies over people’s health, was shown by the extreme inequity in the distribution of vaccines and treatments.

Some countries experienced repercussions from years of neo-liberalism, with insufficient investment in public services (Harvey, 2005). The UK, USA and Belgium struggled with privatized systems, whereas middle-income nations like Vietnam and Thailand, which had previously prioritized public health investments, fared better. The shortcomings of decades of privatization of public services in countries which had embraced neo-liberalism were painfully obvious. Public Service International (Enríquez and Fraga, 2021) noted that the pandemic underlined the essential role of the State and the unwaged social reproduction work of women in attending to the care needs of the population and exercising a leadership role in the social organization of care. The value of universal services was also shown, especially where there was political commitment to a reduction of inequities and a determination to recognize the rights of marginalized groups such as Indigenous peoples, migrants and urban slum dwellers. Taiwan’s success throughout the pandemic (Table 2) and positive economic growth suggests it is possible for a government to protect lives and livelihoods. Taiwan demonstrates how effective intersectoral action can both tackle the pandemic and also protect livelihoods when governments plan in the systematic manner Taiwan was able.

The adoption of radically new equity oriented policies, such as the increase in welfare payments or provision of shelter for homeless people led some civil society actors to see the potential opening of a policy window (Kingdon, 2014) in which these changes could be made permanent. But despite their advocacy the emergency measures were rolled back.

## Conclusion

Pandemics are profoundly political and impact on all sectors of society. Responses to novel infectious diseases require swift action by governments who have to make what may be unpopular decisions and act to restrict the rights of individuals. The quality of their leadership is vital at these times. Good leaders understand the importance of scientific advice and are prepared to put human health above the needs of economic interests. They also need to show compassion and act to protect people in the most vulnerable circumstance. They also need to ensure intersectoral collaboration between government, civil society and the commercial world and between the sectors of governments at all levels. Health, care services, education, social security, economy, development, agriculture, security forces are all involved in tackling a pandemic. Intersectoral collaboration will work best if it exists prior to a pandemic so existing collaborations can be repurposed during the emergency. Recognizing and understanding the importance of the politics of pandemic preparedness is vital for understanding how best to respond to future public health emergencies.

## Data Availability

All data used in this study are publicly available.

## Reflexivity statement

Our group is multidisciplinary, from countries with a variety of national incomes, balanced in terms of gender and levels of research experience as detailed below.

## Multidisciplinary

Our team includes the following disciplinary backgrounds: medicine, public health, epidemiology, psychology, law, social science, economics and political science. In addition the following hold formal positions in the People’s Health Network global or country structures (Baum, De Ceukelaire Falcão, London, Giugliana, Kim, Paremoer), so we bring a strong civil society perspective.

## Country Income Level

Our team includes authors from a low-income country (Ethiopia), middle-income countries (Brazil, India, Thailand, Peru, Vietnam, South Africa) and high-income countries (Australia, Belgium, New Zealand, South Korea, Spain, Taiwan, USA, UK).

## Gender

Our team includes 11 women and 12 men.

## Stage of career

Our authors include the following Early Career Researchers: Musolino, Flavel, Falcão, Gesesew. The following authors are Mid-Career researchers: Freeman, Giugliana, Kim, Nandi, Serag.
